# COMMUNICATION OF LONG-TERM-CARE PLANS AMONG OLDER ADULTS

**DOI:** 10.1093/geroni/igae098.2947

**Published:** 2024-12-31

**Authors:** Charlie Olvera, Amber Miller-Winder, Alaine Murawski, Vanessa Ramirez-Zohfeld, Lee Lindquist

**Affiliations:** Northwestern University Feinberg School of Medicine, Division of Geriatrics, Evanston, Illinois, United States; Northwestern University, Evanston, Illinois, United States; Northwestern University, Evanston, Illinois, United States; Northwestern University, Evanston, Illinois, United States; Northwestern University, Evanston, Illinois, United States

## Abstract

Older Adults (OAs) making long-term care (LTC) plans for post-hospitalization or Alzheimer’s Disease (AD) care must communicate their plans to ensure their wishes are honored. What factors increase the likelihood of this communication? The PlanYourLifespan (PYL) study examines OA LTC decision-making, employing a tool proven effective in helping OAs make LTC decisions. Subjects were administered PYL after baseline assessment, with follow-up assessments every 6 months thereafter. Subjects indicating they made LTC plans were asked if they communicated these plans. We employed GLMM to identify significant (p< 0.05) factors increasing the likelihood of plan communication. 293 subjects enrolled, mean age 73.5, 72.7% female, 40.4% URM. Subjects were more likely to communicate AD LTC plans if they were married (OR2.131), had advanced directives (OR2.004) or living wills (OR1.562), or had higher health literacy (OR2.174). Subjects were more likely to communicate AD in-home care plans if they were married (OR1.871), displayed higher health activation (OR2.425) or health literacy (OR1.883), had advanced directives (OR1.651) or living wills (OR1.596). Subjects who perceived adequate social support (OR2.163), or had advanced directives (OR1.828), living wills (OR1.565), or power of attorney (OR1.529) were more likely to communicate post-hospitalization LTC plans. Married subjects (OR2.034) and those with high health activation (OR1.96) or health literacy (OR1.929) were more likely to communicate post-hospitalization in-home care plans. OAs with higher health literacy and activation, stronger social support, and pre-existing legal directives in place are more likely to communicate LTC plans. These results can help future interventions optimize communication of LTC plans among OAs.

